# Malignant Perivascular Epithelioid Cell Tumor (PEComa) of the Uterus: A Rare Type of Mesenchymal Tumors and a Management Challenge

**DOI:** 10.3390/cancers17132185

**Published:** 2025-06-28

**Authors:** Reyes Oliver-Perez, Marta Ortega, Aranzazu Manzano, Jose Manuel Estrada-Lorenzo, Mario Martinez-Lopez, Elena Zabia, Gregorio Lopez-Gonzalez, Ainhoa Madariaga, Lucia Parrilla, Alvaro Tejerizo, Blanca Gil-Ibañez

**Affiliations:** 1 Gynaecology Oncology Unit, Department of Obstetrics and Gynaecology, University Hospital 12 de Octubre, 28041 Madrid, Spain; gregorio.lopez@salud.madrid.org (G.L.-G.); alvaro.tejerizo@salud.madrid.org (A.T.); blanca.gil@salud.madrid.org (B.G.-I.); 2 Research Institute [imas12], Complutense University of Madrid, 28040 Madrid, Spain; aranzazu.manzano@salud.madrid.org (A.M.); mmartinezlopez2@salud.madrid.org (M.M.-L.); ainhoa.madariaga@salud.madrid.org (A.M.); lucia.parrilla@madrid.salud.org (L.P.); 3 Department of Obstetrics and Gynaecology, University General Hospital of Villalba, 28400 Collado Villalba, Madrid, Spain; marta.ortegab@hgvillalba.es; 4 Department of Medical Oncology, University Hospital 12 de Octubre, 28041 Madrid, Spain; 5 Surest Hospital Library, 28500 Arganda del Rey, Madrid, Spain; josemanuel.estrada@salud.madrid.org; 6 Department of Pathology, University Hospital 12 de Octubre, 28041 Madrid, Spain; 7 Department of Radiology, University Hospital 12 de Octubre, 28041 Madrid, Spain; elena.zabia@salud.madrid.org

**Keywords:** PEComa, mesenchymal neoplasms, mTOR, tuberous sclerosis complex, perivascular epithelioid cell neoplasm

## Abstract

Gynecologic perivascular epithelioid cell tumors (PEComas) are rare mesenchymal neoplasms characterized by the co-expression of melanocytic markers (HMB-45 and Melan-A) and smooth muscle markers (SMA, desmin, and caldesmon). The uterus is the most common organ affected, with approximately 110 cases reported worldwide, while occurrences in the cervix, vagina, ovary, and other gynecologic locations are exceptionally rare. These tumors typically present with nonspecific symptoms such as abnormal uterine bleeding and pelvic pain, often mimicking other uterine neoplasms. Histopathologically, PEComas exhibit epithelioid and spindle cell morphology with variable nuclear atypia, mitotic activity, and characteristic immunohistochemical profiles. Although most PEComas behave benignly, a subset demonstrates malignant potential, associated with larger tumor sizes, an increased mitotic index, necrosis, and vascular invasion; however, standardized diagnostic criteria remain scarce. Molecular alterations frequently involve the mTOR signaling pathway through tuberous sclerosis complex (TSC) 1 and TSC2 gene mutations, offering potential targets for therapy. Surgical resection with clear margins remains the cornerstone of treatment. For advanced or metastatic cases, mTOR inhibitors have shown promising efficacy, whereas the role of radiotherapy remains uncertain. This review aims to synthesize current knowledge regarding the epidemiology, clinical presentation, histologic features, malignant potential, and treatment of uterine PEComas, emphasizing the importance of accurate histopathological classification and molecular profiling to guide individualized therapeutic strategies.

## Simple Summary

Uterine perivascular epithelioid cell tumors (PEComas) represent a rare subset of mesenchymal neoplasms with variable clinical behavior and prognosis. Their low incidence, along with significant histological overlap with other uterine tumors, poses major diagnostic and therapeutic challenges. While many PEComas follow a benign course, some exhibit aggressive behavior. Currently, there are no standardized diagnostic criteria or treatment guidelines. This review provides an updated overview of the epidemiology, clinical presentation, histopathological and immunohistochemical findings, molecular markers, and available treatment strategies, including surgery, mTOR inhibitors, and radiotherapy. The importance of a multidisciplinary approach for accurate diagnosis and individualized management is emphasized. Additionally, the potential of targeted therapies and the prognostic value of histological criteria are discussed to support better clinical decision-making for this rare and complex tumor entity.

## 1. Introduction

Perivascular epithelioid cell tumors (PEComas) are a rare group of mesenchymal neoplasms composed of distinctive perivascular epithelioid cells that co-express both melanocytic and smooth muscle immunohistochemical markers. Initially described in the early 1990s and formally recognized by the World Health Organization in 2002, PEComas were distinguished as a family of tumors that includes angiomyolipoma and lymphangioleiomyomatosis, among others. Since their initial classification, diagnostic criteria have evolved but remain heterogeneous.

Within the gynecologic tract, the uterus is the most frequently affected organ. Due to their low incidence, uterine PEComas represent significant challenges for both diagnosis and clinical management [1,2]. Their rarity, combined with considerable histopathological overlap with other uterine mesenchymal tumors, often complicates accurate classification and may lead to the delayed treatment or misjudgment of their potential for malignancy [3].

Although most uterine PEComas exhibit benign behavior, a subset displays aggressive features. Multiple classification systems have emerged to address this variability, such as the Folpe, Schoolmeester, and Bennet criteria [1,4,5,6,7]. These systems differ in the thresholds and combinations of worrisome features required to designate malignancy or uncertain potential. Their comparative accuracy remains a matter of ongoing debate, particularly in the context of uterine PEComas.

Recent research emphasizes the importance of a more rigorous histopathological evaluation to improve risk stratification and guide clinical decision-making. In addition, molecular alterations—particularly those involving the mTOR signaling pathway—have been increasingly identified in PEComas, offering potential avenues for targeted therapy. Nevertheless, these strategies remain under investigation, and consensus on their clinical application is yet to be established [3].

This review aims to synthesize current knowledge regarding the epidemiology, clinical presentation, histologic and immunophenotypic characteristics, molecular alterations, and prognostic classification systems of uterine PEComas. Specifically, we seek to evaluate the strengths and limitations of existing malignancy stratification models and assess the potential role of mTOR-targeted therapies in individualized patient management. By clarifying diagnostic criteria and highlighting relevant prognostic markers, we aim to support more accurate diagnosis and contribute to the development of individualized treatment approaches for patients with these rare tumors.

## 2. Materials and Methods

A comprehensive literature review was performed. The search strategy included the terms #1 pecoma* OR “perivascular epithelioid cell neoplasm*” OR “Perivascular Epithelioid Cell Neoplasms” [Mesh] #2 uterin* OR ovari* OR gynecol* #3 #1 AND #2, which were searched on PubMED, Cochrane, Web of Science, and EMBASE from inception until February 2025. All references published in English reporting at least one case of uterine PEComa confirmed during pathologic evaluation were included. No selection criteria were applied to the study design or type of publication.

Two independent reviewers (RO and MO) screened the titles and abstracts of the identified references, selected eligible studies, and resolved discrepancies by consensus together with a third reviewer (BG). In cases where the study topic could not be clearly ascertained from the title or the abstract, the full-text version was retrieved for evaluation. The full text of the potentially eligible publications was retrieved and independently assessed for eligibility. An assessment of publication bias was not performed in this study, as all included datasets were retrospective descriptive cohorts without interventional comparisons. A comprehensive review of the selected studies was performed, summarizing the main evidence according to the review sections. The review was not registered on PROSPERO as per the registration policy.

## 3. Results

### 3.1. Epidemiology

The PEComas of the gynecological tract represented approximately 25% of the total of cases reported in the literature, with up to 110 cases of Gynecological PEComas described [2]. The uterine corpus and the uterine cervix are the most common sites of origin, representing 75% and 10% of cases, respectively. The ovary, vagina, and broad ligament are locations rarely described in the literature [8]. The age of patients at presentation ranges from 9 to 79 years, with a peak of incidence falling within the fourth decade of life [2,8].

Most PEComas are sporadic. However, approximately 8–10% are associated with tuberous sclerosis complex (TSC), an autosomal dominant syndrome characterized by a wide variety of neoplastic manifestations (renal angiomyolipomas, lymphangioleiomyomatosis, cardiac rhabdomyomas, and subependymal giant cell astrocytomas, among others) [5,9]. This association is due to genetic mutations that result in the inactivation of the TSC1 (9q34) or TSC2 (16p13.3) genes, leading to the impaired production of proteins hamartin and tuberin, respectively [1,5,10]. TSC1 and TSC2 interact as heterodimers to inhibit the mammalian target of the rapamycin (mTOR) pathway; therefore, their inactivation leads to increased cell growth and proliferation [5,11]. TSC is frequently characterized by multiple PEComas with more malignant behavior and the possible difficulty in discriminating between recurrence and a second primary PEComa [7,11]. In fact, most cases of PEComatosis, defined as widespread multifocal macroscopic and microscopic nodules of the PEComa cells involving multiple sites in the gynecological tract and pelvis, occur in TSC patients [5].

### 3.2. Clinical Presentation, Tumor Markers, and Differential Diagnosis

Uterine PEComas often present with nonspecific and insidious symptoms, which can contribute to delays in diagnosis. The most common clinical manifestations include abnormal uterine bleeding, pelvic or abdominal pain, and, less frequently, the identification of a palpable pelvic mass upon physical examination. Some tumors are incidentally identified during routine gynecologic procedures or imaging studies performed for unrelated indications. Rarely, acute presentations such as uterine rupture or hemoperitoneum have also been reported, particularly in pregnant women or those with large or rapidly growing tumors [1,2,12,13].

No specific serum tumor markers have been consistently associated with PEComas, limiting their applicability in diagnosis or follow-up. In isolated cases, elevated CA-125 levels have been observed, but this finding is nonspecific and may reflect peritoneal irritation or concurrent pathology [14].

The differential diagnosis is broad and includes other mesenchymal neoplasms of the uterus, such as leiomyomas, leiomyosarcomas, endometrial stromal sarcomas, and undifferentiated uterine sarcomas. Given the clinical and macroscopic similarities among these entities, accurate diagnosis is often challenging. Diagnostic ambiguity is compounded by the absence of standardized histopathological criteria, so reliance on nonspecific clinical features can lead to uncertainty. Clinical suspicion may arise in cases of rapidly enlarging uterine masses or atypical presentations, but the unpredictable benign vs. malignant behavior of PEComas can create clinical urgency that requires prompt and careful evaluation [3,4,15].

### 3.3. Imaging Features

There are no imaging techniques or characteristics that can distinguish PEComas from other benign or malignant gynecological tumors, such as leiomyoma or leiomyosarcoma. The lack of specific radiological findings makes the diagnosis and preoperative management of these tumors challenging. The scarcity of case reports suggests that these lesions typically present as well-circumscribed solid masses, with homogenous solid components intermixed with areas of necrosis or hemorrhage and, in some cases, intratumoral fat (Table 1) [10,16,17,18,19,20,21].

Malignant PEComas generally present as large tumors with a tendency for aggressive growth, although they typically remain well-circumscribed compared to other malignant uterine tumors. Lymph node involvement is unusual in uterine PEComas, but its presence could raise suspicion for malignancy. If metastatic disease is present, it usually involves the peritoneum, lungs, or liver [16,17,18].

**Figure 1:**
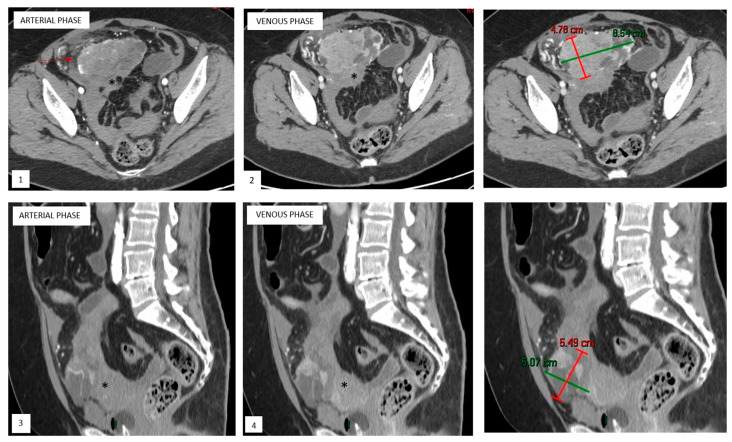
Axial and sagittal reformatted contrast-enhanced CT pelvic image of ruptured uterine PEComa with hemoperitoneum. Axial and sagittal reformatted contrast-enhanced CT pelvic images reveal a well-marginated mass arising exophytically from the uterine fundus (*), with hypoattenuating areas consistent with cystic/necrotic changes and solid components showing progressive heterogeneous enhancement during arterial ((**1**,**3**)) and venous ((**2**,**4**)) phases. A small amount of peritoneal fluid can also be noted (arrow).

Upon computed tomography (CT) (Figure 1), uterine PEComas typically appear as iso- to hypodense masses relative to the surrounding myometrium on non-contrast images. Additionally, internal hemorrhage can be seen as hyperdense areas [16,22].

Magnetic resonance imaging (MRI) is also nonspecific. On T1-weighted sequences, these tumors typically show hypo- to isointensity compared to skeletal muscle. The areas of hyperintensity may indicate hemorrhage or fat. On T2-weighted sequences, PEComas tend to be hyperintense, likely due to the presence of cystic or necrotic areas within the tumor [16,17,21,22]. Fat suppression sequences can help identify intratumoral fat. Restricted diffusion may be present in malignant PEComas. Post-contrast CT and MRI images demonstrate heterogeneous and pronounced enhancement, reflecting their rich vascular supply [16,17,21,22].

Regarding ultrasound, no pathognomonic pattern for PEComas has been described in the literature. Therefore, uterine PEComas are easily misdiagnosed as uterine fibroids, uterine smooth muscle tumors of uncertain malignant potential, or sarcomas. However, most documented cases of uterine PEComas present as a single uterine tumor, distinct from the surrounding myometrium. Their size varies widely (mean diameter around 4 cm; range of 1–10 cm) [16]. They exhibit heterogeneous echogenicity, a high central vascular network with low impedance, and an imprecise tumor border [10,13,16,18,22]. Some authors describe hypoechoic/anechoic areas within the tumor, without significant shadowing or calcification, which may lead to the diagnosis of degenerate leiomyoma or leiomyoma [10,13,16,18].

In summary, although there is no available evidence about the diagnostic accuracy of the mentioned imaging techniques in the literature, gynecologic ultrasound should be the initial diagnostic modality in the evaluation of uterine lesions due to its accessibility. If further assessment is needed, MRI should be the preferred option, as it offers a superior characterization of uterine pathology compared to CT.

### 3.4. Pathologic, Immunohistochemical, and Molecular Features

Macroscopically, PEComas may present as polypoid or pedunculated masses protruding into the endometrial cavity, although they are more commonly based within the myometrium [23].

**Figure 2:**
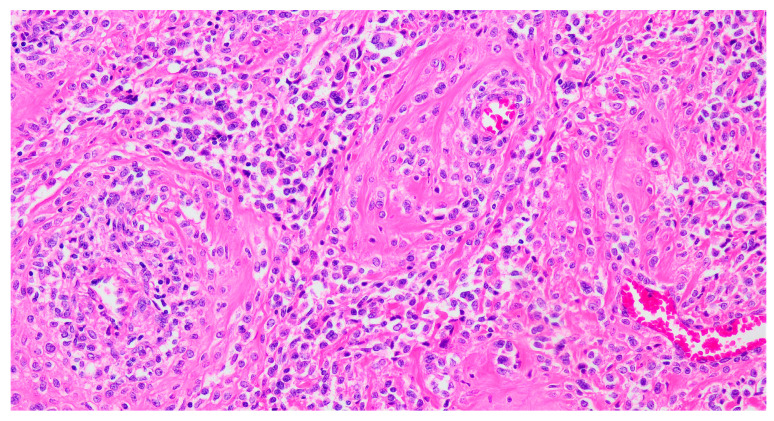
Hematoxylin–eosin-stained section from a uterine PEComa. Epithelioid cells with clear to eosinophilic cytoplasm in a radial/perivascular distribution, with stromal hyalinization (20× magnification).

Histologically, PEComas are composed of epithelioid cells and, less frequently, spindle cells. Epithelioid cells exhibit clear to eosinophilic granular cytoplasm and are typically arranged in dyscohesive nests surrounded by delicate, thin-walled blood vessels and/or in solid sheets (Figure 2). In contrast, spindle cells often form fascicles. Additional features may include perivascular distribution, multinucleated cells, lipid-rich or rhabdoid cytoplasm, and stromal hyalinization. Tumors may display expansile, permeative, or infiltrative growth patterns. Both cell types can show variable degrees of cytological atypia and mitotic activity, and melanin pigment may also be present [24].

**Figure 3:**
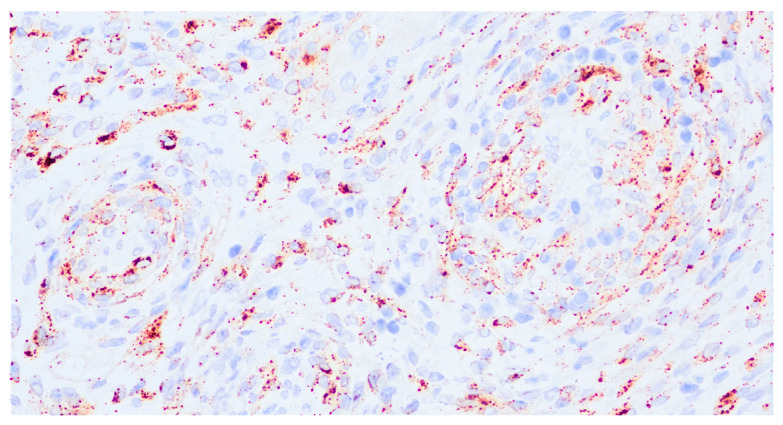
Immunostaining of HMB-45 in a uterine PEComa. Patchy granular cytoplasmic staining for HMB-45 (40× magnification).

PEComas are characterized by the immunoexpression, among others, of both melanocytic markers (HMB-45, Melan-A, MiTF, and PNL2) and myoid markers (desmin, smooth muscle actin, muscle-specific actin, myosin, and calponin). HMB-45 (more specific) and Cathepsin K (more sensitive) are expressed in virtually all PEComas, ranging from diffuse staining to isolated positive cells (Figure 3). Melan-A staining and MiTF are expressed with variable intensity in more than two-thirds of cases, and they are usually focal and less extensive than HMB-45 [6]. MiTF tends to be weak and lacks specificity, and PNL2 shows variable cytoplasmic expression. Except for TFE3-rearranged tumors, the expression of at least one smooth muscle marker is typically strongly positive (SMA being the most expressed). Overall, melanocytic markers are predominantly expressed in epithelioid cells, while myogenic markers are more commonly seen in spindle cells, but overlaps may occur [15].

Molecular alterations in conventional PEComas often involve mutations, copy number losses, or rearrangements in the TSC1 or TSC2 genes, and they are reported in up to 90% of cases. A smaller subset of PEComas harbors rearrangements involving TFE3 or RAD51B [4]. Accurate diagnosis is essential given the potential for targeted therapy with mTOR inhibitors [25].

### 3.5. Prognosis and Malignant Potential. Risk Stratification Systems

The malignant potential and prognostic evaluation of gynecologic PEComas, particularly those arising in the uterus, remain a significant clinical challenge.

In a retrospective analysis of 101 cases of uterine PEComas, Garzon et al. reported a recurrence rate of 34.7% following first-line therapy, with a median time to recurrence of 9.5 months and a median follow-up of 18 months [7]. Notably, only 8.9% of patients presented with advanced or metastatic disease at the time of diagnosis. Similarly, Jiang et al. highlighted a substantial risk of recurrence and/or metastasis in gynecologic PEComas, reporting recurrence and mortality rates of 31.1% and 6.5%, respectively [8]. Nevertheless, given the rarity of the disease, the potential for aggressive clinical behavior—particularly among cases initially diagnosed as non-metastatic—remains an unresolved clinical question.

Five histopathological features have been associated with malignant potential: tumor size greater than or equal to 5 cm, high-grade nuclear atypia, mitotic rate greater than or equal to 1/50 high-power fields (HPF), tumor necrosis, and lymphovascular invasion [5,6,7]. To date, no differences in the prognosis of PEComas have been demonstrated based on their immunohistochemical profile [8].

Based on the combinations of these worrisome features, five classifications have been proposed to predict PEComa behavior: the original Folpe criteria, modified Folpe criteria, Schoolmeester criteria, Bennet criteria, and the criteria referenced in the recent World Health Organization’s (WHO) classification [5,6,7,14,19]. These systems differ in the specific combinations and thresholds of features required to classify a tumor as benign, malignant, or as having uncertain malignant potential (Table 2).

The original 2005 Folpe criteria, based on a retrospective analysis of 26 PEComas of various sites, classified these tumors into benign (no worrisome features), uncertain malignant potential (either tumor size greater than 5 cm or nuclear pleomorphism/multinucleated giant cells), and malignant (two or more worrisome features) (Table 2) [15]. While widely used, studies have shown that the Folpe criteria only achieved an accuracy of 69–71% in predicting malignant behavior, with a high false-positive rate of up to 30% [5,8]. Furthermore, its applicability to uterine PEComas has been questioned, partly because only 4 of the 26 cases included in the analysis had a uterine origin.

The modified Folpe criteria (Table 2), wherein tumors with only one risk factor are considered benign, may be more accurate in the context of PEComas of the gynecological tract. Due to the lack of available data, in this system, tumors with isolated marked atypia, maximum dimension of >10 cm, or mitotic rate ≥ 4/50 HPF in the absence of other worrisome criteria should be considered with uncertain malignant potential [5].

Based on a study of metastatic uterine PEComas, Schoolmeester et al. modified the classification and suggested a higher threshold for malignancy [1]. This system classifies tumors with four or more worrisome features as malignant and those with fewer than four as benign or uncertain (Table 2). By increasing the threshold, the Schoolmeester criterion significantly improved the specificity in predicting malignant behavior. However, since all cases included in this study were metastatic PEComas, this led to a false-negative rate of up to 14%. [1,5,8]. In this context, the Bennet criteria represent a modification of the Schoolmeester system, defining malignancy based on the presence of three or more specific worrisome features [6].

In a comparative study of these four classifications, focusing on uterine PEComas without TSC association, the modified Folpe criteria demonstrated the highest hazard ratio (HR) for recurrence (HR: 8.63; 95% confidence interval [CI] 2.06–36.1) and death (HR: 6.8, 95% CI: 0.89–51.6), suggesting superior prognostic accuracy [7]. The Schoolmeester criteria did not show a statistically significant difference in survival outcomes between groups, while the Bennet criteria showed a statistically significant HR for death (HR 4.3, 95% CI: 1.22–15.2), though it was lower than the modified Folpe criteria [7]. Some authors proposed the further refinement of the modified Folpe criteria by increasing the thresholds for tumor size (≥8 cm) and mitotic activity (≥5 mitoses per 50 HPF), which may enhance prognostic accuracy [7].

Finally, in the most recent WHO classification of soft tissue tumors, PEComas characterized by varying combinations of mitotic activity, necrosis, and pleomorphism are categorized as malignant (Table 2). In a comparative analysis, Jiang et al. reported similar accuracy but a much higher false-negative rate for this classification compared to the Schoolmeester criteria [8]. However, the prognostic validity and clinical utility of this classification, specifically in gynecologic PEComas, remains unestablished [24].

In summary, while the modified Folpe classification currently provides the most reliable prognostic stratification for uterine PEComas, the development of a universally validated and clinically applicable system remains critical. Future research should prioritize the incorporation of immunohistochemical markers, such as MELAN-A and hormone receptors, along with molecular profiling, to refine risk assessment and guide therapeutic strategies [7].

### 3.6. Treatment

(a) Surgery: Although there is no established optimal surgery strategy, complete tumor resection with negative margins appears to be the cornerstone of surgical treatment [2].

Hysterectomy is the most frequent surgical strategy reported in the case series published in the literature, but there are cases of mass resection alone without a worse prognosis [26]. Therefore, if PEComa is suspected or diagnosed preoperatively, the decision between mass resection or hysterectomy will depend on the tumor´s location and the patient´s desire for fertility preservation [2,27]. In cases where the histopathological diagnosis is made after surgery with only tumor resection and clear margins, reoperation to perform a hysterectomy does not appear to be necessary, especially when the histopathology indicates benign features, although long-term follow-up is recommended [28]. Salpingo-oophorectomy is indicated when there is a high risk of malignancy or when the tumor involves the adnexa to ensure complete resection and minimize the risk of recurrence [2,17]. Lymphadenectomy appears to be controversial and not supported [7].

(b) Systemic treatment: PEComas are considered relatively chemoresistant neoplasms [29]. In cases of localized disease, there is insufficient evidence for recommending adjuvant therapy following initial surgery [30]. Since there are no large clinical trials due to the rarity of these tumors, therapeutic decisions are based on case reports and small series. Adjuvant treatment can be considered in high-risk cases. For patients with locally advanced or metastatic disease, molecular targeted therapy has become the mainstay of systemic treatment, guided by the tumor’s underlying molecular profile.

The single-arm phase II AMPECT trial demonstrated the activity of nab-sirolimus (nanoparticle albumin-bound sirolimus), a next-generation mTOR inhibitor, with an overall response rate (ORR) of 38.7% (95% confidence interval—CI: 21.8–57.8) and a median progression-free survival (PFS) of 10.6 months (95% CI: 5.5–41.2). Notably, responses were durable, with a median duration of response of 39.7 months (95% CI: 6.5–not estimable) [31]. Other mTOR inhibitors, including everolimus, temsirolimus, and sirolimus, have shown similar efficacy, with ORRs around 40% in retrospective series, and they are considered the standard first-line treatment options [32]. The clinical benefit appears to be higher in tumors harboring TSC2 mutations. In contrast, PEComas with TFE3 rearrangements, associated with MET pathway activation, tend to exhibit lower response rates to mTOR inhibitors [1,31].

Case reports and small retrospective series have documented the modest activity of hormonal therapies—either as monotherapy or combined with mTOR inhibitors—as a strategy to overcome resistance to targeted agents [32,33]. Additional strategies under investigation include combinations with antiangiogenic agents [34]. When targeted therapies are unavailable or ineffective, alternative treatment options include antiangiogenic agents such as pazopanib, sunitinib, or sorafenib [35]. Cytotoxic chemotherapy with gemcitabine-based regimens (e.g., combined with dacarbazine or docetaxel), anthracyclines, or ifosfamide may be employed in later lines, albeit with modest clinical benefit [29].

Despite emerging data, only nab-sirolimus has received approval by the U.S. Food and Drug Administration, granted in 2021 for the treatment of locally advanced or metastatic malignant PEComas regardless of molecular status. The European Medicines Agency has not approved any specific treatments for PEComas, although access through compassionate use programs may be available depending on the country.

Although not well established in PEComa, the known mechanisms of resistance to mTOR inhibitors in other tumors include the activation of the PI3K/AKT or MAPK/ERK pathways, mutation in the kinase domains of TSC1/TSC2, overexpression of ABC transporters, MET activation, or microenvironment changes. Future strategies to overcome resistance may involve the use of dual PI3K/AKT inhibitors or combination therapies with antiangiogenics, immunotherapy, or hormone therapy, as previously mentioned. These approaches warrant further investigation [36]:

(c) Radiation therapy: The role of radiotherapy (RT) in the management of uterine PEComas is controversial. RT may be considered in selected cases, such as incomplete excision (positive margins), local recurrence, or high-grade tumors [2,3].

The available literature on RT in PEComas is limited and consists largely of case reports and small series, with variable outcomes. While some cases have reported local disease control following RT, its overall efficacy remains uncertain. Recent publications have highlighted the limited radiosensitivity of PEComas and the lack of standardized treatment indications [37,38].

Nevertheless, emerging evidence suggests a potential role for RT within multimodal treatment strategies. For instance, stereotactic body radiotherapy (SBRT) in combination with immunotherapy agents such as PD-1 inhibitors and granulocyte–macrophage colony-stimulating factor (GM-CSF) has shown promising results in advanced or unresectable cases, although these remain preliminary observations [39]. Therefore, some authors suggest that RT may play a palliative role in controlling symptoms such as pain or bleeding in patients with unresectable or metastatic disease [38].

## 4. Discussion

The present review summarizes the available evidence on uterine PEComas. These tumors represent a rare group of mesenchymal neoplasms composed of epithelioid and spindle cells with myomelanocytic differentiation. PEComas are frequently benign and incidentally discovered after surgical resection. However, approximately 35% exhibit aggressive behavior, including local recurrence or distant metastasis, with reported recurrence and mortality rates of 31.1% and 6.5%, respectively [8]. Nevertheless, due to their low incidence—only about 110 cases reported worldwide in the English-language literature—significant challenges remain regarding both diagnosis and risk stratification, making the clinical management of these tumors particularly challenging.

Uterine PEComas are often misdiagnosed and, in most cases, are only identified after pathological examination. These tumors typically present with nonspecific and insidious symptoms, such as abnormal uterine bleeding and pelvic pain, often mimicking other uterine tumors [1,2]. This resemblance can lead to conservative management, despite the fact that some PEComas may exhibit malignant behavior.

Due to their heterogeneous imaging characteristics, PEComas can resemble various other uterine lesions, including well-demarcated tumors such as typical leiomyomas, as well as more heterogeneous tumors like atypical or degenerated leiomyomas, smooth muscle tumors of uncertain malignant potential (STUMPs), or other uterine sarcomas. Some authors consider certain features—such as solitary uterine tumors with heterogeneous echogenicity and central vascularization—as diagnostic clues for PEComas [10,13,16,18,22]. However, the reality is that neither preoperative ultrasound, MRI, nor CT scans are able to reliably differentiate PEComas from these other tumors [7,10]. This limitation can significantly affect both the choice of the surgical approach and the overall prognosis.

For these tumors, surgical resection with clear margins is considered the cornerstone of treatment [2,7]. However, subsequent management strategies for PEComa remain undefined. Adjuvant treatment appears to be necessary in high-risk patients [7]. Nevertheless, the malignant potential of PEComas remains uncertain due to the rarity of this entity and the associated limitations in conducting studies.

Both benign and malignant PEComas share certain immunohistochemical features; therefore, common markers such as HMB-45 and Melan-A do not differentiate between benign and malignant forms [8]. As a result, efforts to define malignancy criteria have focused on the histopathological characteristics of the primary tumor.

Based on specific combinations and thresholds of worrisome histopathological features, five different risk stratification systems have been proposed: Folpe, modified Folpe, Bennet, Schoolmeester, and WHO classifications [1,5,6,7,24]. However, since all of them are based on retrospective series of PEComas from various origins, none have demonstrated high accuracy in predicting malignant behavior specifically in gynecologic PEComas.

The Folpe criteria [15] are the most widely used system. However, some deficiencies have become apparent. While categorizing cases with no worrisome features as benign and those with two or more worrisome features as malignant is direct, it remains unclear how PEComas with a single worrisome feature should be classified, such as necrosis, infiltrative growth pattern, or elevated mitotic count [5,15]. Furthermore, its applicability to uterine PEComas has been questioned, partly because only 4 of the 26 cases included in the analysis originated in the uterus. Schoolmeester et al. [1] raised the threshold for diagnosing malignant behavior by defining PEComas with four or more worrisome features as malignant in the context of 16 metastatic uterine PEComas. However, its strict focus on metastatic uterine PEComas may significantly limit its application to all PEComas, especially those without extrauterine disease. Conlon et al. [5] proposed the modified Folpe criteria, which appear to have the highest accuracy, although this classification has not been validated. The recent WHO classification [24], modified for gynecologic tumors, may improve the accuracy of these systems; however, its prognostic validity has not been established either.

Therefore, to date, there is no standardized and universally adopted risk classification for PEComas—particularly gynecological PEComas—that has demonstrated high efficacy in accurately predicting the malignancy of these tumors. Furthermore, there is ongoing controversy among the four reported classification systems, which means that a subset of PEComas may be classified as malignant or not depending on the system used. Likewise, all existing systems rely exclusively on histopathological features, the evaluation of which is examiner-dependent and may be limited by interobserver variability. In this context, with the aim of standardizing criteria for adjuvant treatment, it is imperative to improve and unify the classification systems for uterine PEComas—ideally through multicenter studies. Along these lines, additional immunohistochemical or genetic markers may be incorporated to refine risk assessment and guide therapeutic strategies for these tumors.

Data on postoperative management remain inconclusive. Adjuvant treatment—mainly chemotherapy with or without radiotherapy—was used in a minority of cases, employing heterogeneous chemotherapeutic agents, and it appears to be largely ineffective in advanced disease. This precludes drawing any definitive conclusions regarding the most appropriate treatment protocol. Molecular targeted therapy has emerged as the mainstay of systemic treatment, guided by the tumor’s underlying molecular profile [1,32]. In this context, targeting the mTOR pathway—which is upregulated in PEComas—has shown promising results [32]. Therefore, molecular profiling is mandatory to guide targeted therapies.

The lack of consensus regarding the treatment of gynecological PEComas is due to several factors, including the small number of cases reported in the literature and the absence of randomized studies. In this regard, beyond reaching consensus on criteria for additional treatment, establishing a prospective multicenter registry of these cases is crucial to improving the knowledge and management of patients with malignant PEComas.

This work has several limitations. First, only the literature published in English was reviewed, so cases reported in other languages may have been missed. Second, as a comprehensive review and retrospective study, we were limited to summarizing data reported in the identified studies; therefore, potentially relevant information may have been excluded, and no quantitative analysis was performed.

## 5. Conclusions

Uterine PEComas represent significant challenges for both diagnosis and clinical management. Despite their low incidence, due to their nonspecific clinical presentation and potentially aggressive behavior, these tumors should be included in the differential diagnosis of atypical uterine lesions. The development of a universally validated and clinically applicable prognostic stratification system remains critical for improving the management of these tumors and highlights the need for further investigation in this context. The variety of therapeutic strategies used—with heterogeneous results—along with the lack of established guidelines, highlights the need for large-scale, multicenter studies to unify and validate risk stratification systems and treatment protocols.

## Figures and Tables

**Table 1:** Imaging features of PEComas, leiomyoma, and leiomyosarcoma.

	PEComa	Leiomyoma	Leiomyosarcoma
US	Single uterine tumor. Heterogeneous echogenicity (presence of hypoechoic/anechoic areas). Imprecise or regular tumor border. Absent acoustic shadowing. High central vascular network.	Single or multiple tumors. Homogeneous echogenicity. Regular border. Acoustic shadowing. Peripheral vascularity	Nonuniform hypoechogenic mass. Undefined border. Absent acoustic shadowing. Frequent peripheral and internal vascularity.
CT	Well-defined mass. Heterogenous density. Iso- to hypodense relative to muscle. Hyperdense areas if hemorrhage is present. Arterial phase enhancement.	Well-defined mass. Iso- or hypoattenuating Coarse or diffuse calcification can be present. Low definition.	Large and irregular mass. Heterogenous density. Presence of central necrosis and hemorrhage areas. Calcifications are rare. Distant metastases.
MRI	Well-defined mass. T1-weighted: hypo- to isointense compared to skeletal muscle. T2-weighted: hyperintense. DWI: restricted diffusion if malignant. Possible presence of necrosis, hemorrhage, or fat.	Well-defined mass. T1-weighted: isointense or hypointense relative to myometrium. T2-weighted: hypointense relative to myometrium. DWI: No restricted diffusion.	Large and irregular mass. Heterogeneous signal intensity in T1- and T2-weighted images. Hemorrhagic and central necrosis areas. DWI: restricted diffusion.

US, Ultrasound; CT, computed tomography; MRI, magnetic resonance imaging; DWI, diffusion-weighted imaging.

**Table 2:** Summary of risk stratification systems for gynecological PEComas.

Classification System	Benign	Uncertain Malignant Potential	Malignant
Folpe criteria	No worrisome features: ▪ Size < 5 cm ▪ Non-infiltrative growth pattern ▪ Non-high nuclear grade and cellularity ▪ Mitotic rate < 1 per 50 HPF ▪ No necrosis ▪ No vascular invasion	One or both worrisome features: ▪ Size > 5 cm ▪ Nuclear pleomorphism/multinucleated giant cells	Two or more worrisome features: ▪ Size > 5 cm ▪ Infiltrative growth pattern ▪ High nuclear grade and cellularity ▪ Mitotic rate ≥ 1 per 50 HPF ▪ Necrosis ▪ Vascular invasion
Modified Folpe criteria	One or no worrisome features: ▪ Size ≥ 5 to < 10 cm ▪ Infiltrative growth pattern ▪ Mitotic rate of 2–3 per 50 HPF ▪ Lymphovascular invasion ^a^	One worrisome feature: ▪ Size ≥ 10 cm ▪ Isolated marked atypia ▪ Mitotic rate ≥ 4 per 50 HPF	Any necrosis or more than two worrisome features: ▪ Size ≥ 5 cm ▪ Marked atypia ▪ Mitotic rate > 1 per 50 HPF ▪ Lymphovascular invasion
Schoolmeester criteria	Fewer than four worrisome features: ▪ Size ≥ 5 cm ▪ High nuclear grade ▪ Necrosis ▪ Mitotic rate ≥ 1 per 50 HPF ▪ Lymphovascular invasion		Four or more worrisome features: ▪ Size ≥ 5 cm ▪ High nuclear grade ▪ Necrosis ▪ Mitotic ≥ 1 per 50 HPF ▪ Lymphovascular invasion
Bennet Criteria	-	Fewer than three worrisome features: ▪ Size ≥ 5 cm ▪ High nuclear grade and cellularity ▪ Mitotic rate ≥ 1 per 50 HPF ▪ Necrosis ▪ Lymphovascular invasion	Three or more worrisome features: ▪ Size ≥ 5 cm ▪ High nuclear grade and cellularity ▪ Mitotic rate ≥ 1 per 50 HPF ▪ Necrosis ▪ Lymphovascular invasion
WHO criteria ^b^	-	Fewer than three worrisome features: ▪ Size ≥ 5 cm ▪ High nuclear grade ▪ Mitotic rate ≥ 1 per 50 HPF ▪ Necrosis ▪ Lymphovascular invasion	Three or more worrisome features: ▪ Size ≥ 5 cm ▪ High nuclear grade and cellularity ▪ Mitotic rate ≥ 1 per 50 HPF ▪ Necrosis ▪ Lymphovascular invasion

HPF, High-power fields ^a^ Caution should be exercised when evaluating tumours exhibiting LVI as the sole worrisome feature, as outcome data in such cases are very limited. ^b^ Modified gynaecology-specific criteria.

## Abbreviations

PEComas: Perivascular epithelioid cell tumors [PEComas]; TSC, tuberous sclerosis complex; TC, computed tomography; MRI, magnetic resonance imaging [MRI]; HPF, high-power field; WHO, World Health Organization; ORR, overall response rate; PFS, median progression-free survival; RT, radiotherapy.
